# Supplementary material for: The Construction and Exploration of a Comprehensive MicroRNA Centered Regulatory Network in Foxtail Millet (Setaria italica L.)
Source: Front Plant Sci. 2022 May 6;13:848474. doi: 10.3389/fpls.2022.848474 (PMC9121102; doi:10.3389/fpls.2022.848474)
Supplement: Supplementary file 1 [file Data_Sheet_1.zip › Sit-miR395c_Seita.8G095800.1_501_TPlot.pdf]

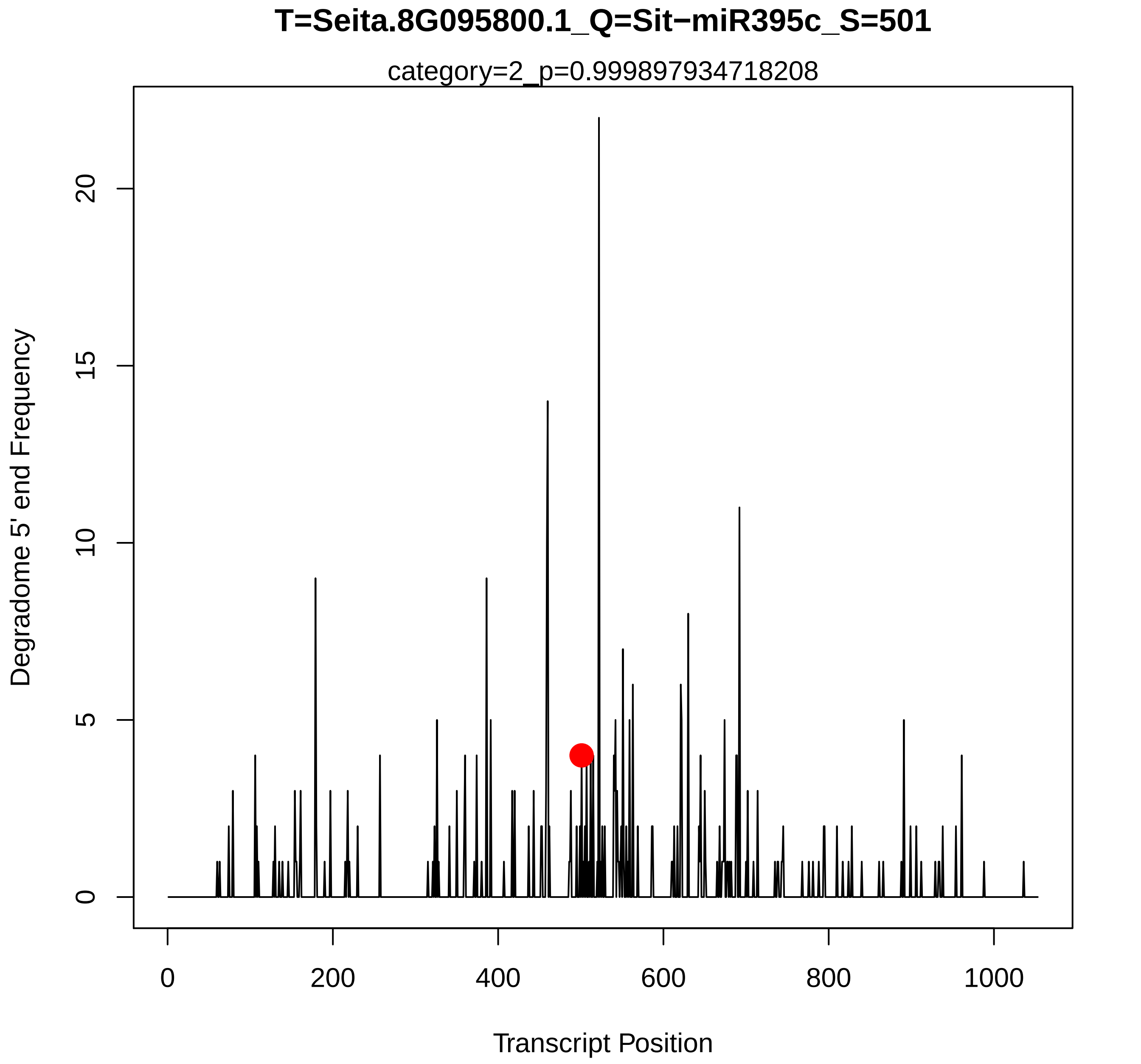
T=Seita.8G095800.1_Q=Sit−miR395c_S=501
category=2_p=0.999897934718208
Degradome 5' end Frequency
0
5
10
15
20
0
200
400
600
800
1000
Transcript Position
